# Therapeutic Potential of Curcumin for the Treatment of Brain Tumors

**DOI:** 10.1155/2016/9324085

**Published:** 2016-10-11

**Authors:** Neil V. Klinger, Sandeep Mittal

**Affiliations:** ^1^Department of Neurosurgery, Wayne State University, Detroit, MI, USA; ^2^Department of Oncology, Wayne State University, Detroit, MI, USA; ^3^Karmanos Cancer Institute, Wayne State University, Detroit, MI, USA

## Abstract

Brain malignancies currently carry a poor prognosis despite the current multimodal standard of care that includes surgical resection and adjuvant chemotherapy and radiation. As new therapies are desperately needed, naturally occurring chemical compounds have been studied for their potential chemotherapeutic benefits and low toxicity profile. Curcumin, found in the rhizome of turmeric, has extensive therapeutic promise via its antioxidant, anti-inflammatory, and antiproliferative properties. Preclinical* in vitro* and* in vivo* data have shown it to be an effective treatment for brain tumors including glioblastoma multiforme. These effects are potentiated by curcumin's ability to induce G2/M cell cycle arrest, activation of apoptotic pathways, induction of autophagy, disruption of molecular signaling, inhibition of invasion, and metastasis and by increasing the efficacy of existing chemotherapeutics. Further, clinical data suggest that it has low toxicity in humans even at large doses. Curcumin is a promising nutraceutical compound that should be evaluated in clinical trials for the treatment of human brain tumors.

## 1. Introduction

Primary central nervous system tumors have an incidence of 27.86 per 100,000 [1]. Glioblastoma (GBM) is the most common malignant primary CNS tumor with an annual incidence of 3.19 per 100,000. GBM accounts for 15.4% of all primary brain tumors and 45.6% of primary malignant brain tumors [1, 2]. The current standard of care for GBM consists of surgical resection followed by the Stupp regimen (adjuvant chemotherapy and radiation) and it has been used in practice for over a decade without substantial improvements. This aggressive multimodal treatment prolongs median overall survival to 15 months [3–5]. Other therapies approved for use in GBM include alternating electrical fields produced by Optune Therapy (Novocure Inc.), further prolonging median survival to 20 months [6]. With such poor prognoses, additional therapeutics are needed to improve survival and quality of life for patients with malignant gliomas. Complementary medicine and nutraceuticals are of particular interest and have been studied extensively as anticancer agents because they are usually associated with low toxicity profiles. This allows them to be safely used at high doses or added to existing chemotherapy regimens as adjuvant treatment.

**Figure 1:**
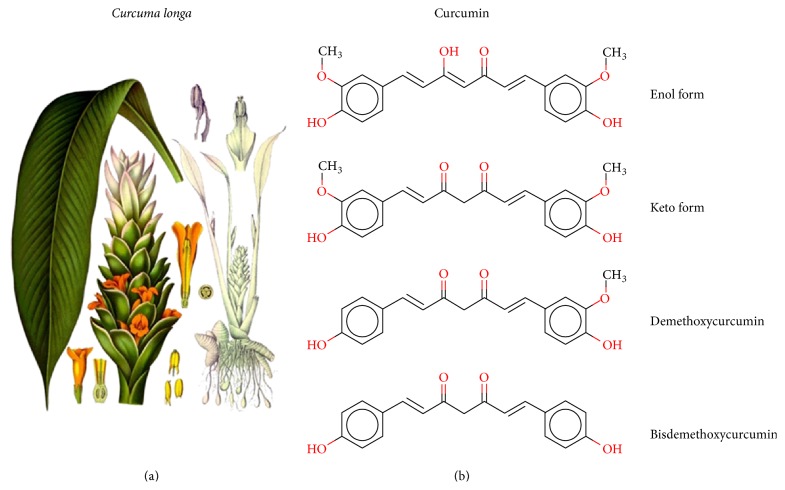
(a)* Curcuma longa* from Koehler's Medicinal Plants, 1887. (b) From top to bottom, curcumin in its enol form, curcumin in its keto form, demethoxycurcumin, and bisdemethoxycurcumin.

Curcumin is found in rhizome of turmeric (*Curcuma longa*), which is a member of the ginger family (Figure 1) [7]. Its traditional uses, long known by Ayurvedic medicine from India and traditional Chinese medicine, include treating infections, liver and skin disorders, dressing wounds, burns, and decreasing inflammation [7]. In fact, powdered* Curcuma longa* has been used in Asian medicine, cosmetics, and fabric dying for more than 2000 years [8]. Curcumin possesses multiple beneficial chemical properties, including antioxidant, anti-inflammatory, and chemotherapeutic potential both against cultured cells and in treatment studies using animal models. Current data suggests that curcumin may be useful in a wide spectrum of human disorders including Alzheimer's disease, Parkinson's disease, diabetes, cardiovascular disease, arthritis, and various neoplasms including brain tumors [9, 10].

## 2. Mechanism and Preclinical Data

**Figure 2:**
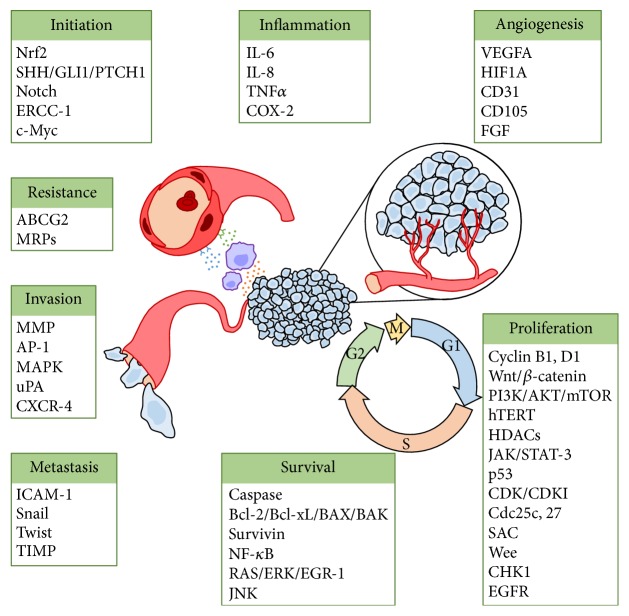
Cancer pathway targets affected by curcumin. When the listed targets have multiple contributions to tumorigenesis (e.g., NF-*κ*B), they are only placed under one category. Figure modified^*∗*^ from Davis et al. 2003 [44]. ^*∗*^As permitted by Creative Commons Attribution-NonCommercial-Share Alike 3.0 license and BJC OPEN initiative. ABCG2: ATP-binding cassette subfamily G member 2; AKT: protein kinase B; AP-1: activator protein 1; BAK: Bcl-2 homologous antagonist/killer; BAX: Bcl-2-like protein 4; Bcl-2: B-cell lymphoma 2; Bcl-xL: B-cell lymphoma-extra large; caspase: cysteine-aspartic protease; CD105: endoglin; CD31: platelet endothelial cell adhesion molecule (PECAM-1); CDC: cell division cycle; CDK: cyclin-dependent kinase; CDKI: cyclin-dependent kinase inhibitor; CHK1: checkpoint kinase 1; c-Myc: v-Myc avian myelocytomatosis viral oncogene homolog; COX-2: prostaglandin-endoperoxide synthase 2; CXCR-4: C-X-C chemokine receptor type 4; EGFR: epidermal growth factor receptor; EGR-1: early growth response protein 1; ERCC-1: excision repair cross-complementation group 1; ERK: extracellular signal-regulated kinases; FGF: fibroblast growth factor; GLI1: GLI family zinc finger 1; HDAC: histone deacetylases; HIF1A: hypoxia inducible factor 1, alpha subunit; hTERT: telomerase reverse transcriptase; ICAM-1: intercellular adhesion molecule 1; IL: interleukin; JAK: Janus kinase; JNK: c-Jun N-terminal kinase; MAPK: mitogen-activated protein kinases; MMP: matrix metalloproteinases; MRP: multidrug resistance protein; mTOR: mechanistic target of rapamycin; NF-*κ*B: nuclear factor kappa-light-chain-enhancer of activated B cells; Nrf2: nuclear factor (erythroid-derived 2) like 2; PI3K: phosphatidylinositol-4,5-bisphosphate 3-kinase; PTCH1: patched 1; SAC: spindle assembly checkpoint; SHH: sonic hedgehog; Snail: zinc finger protein SNAI1; STAT-3: signal transducer and activator of transcription 3; TIMP: tissue inhibitors of metalloproteinases; TNF: tumor necrosis factor; uPA: urokinase; VEGF: vascular endothelial growth factor.

Curcumin's effects on glioma cells* in vitro* and* in vivo* have been well studied. Curcumin has many molecular targets (Figure 2) and therefore diverse and complex mechanisms of action. The antitumoral effects of curcumin are thought to act through many different signaling pathways such as cellular proliferation [11–13], apoptosis [11, 12, 14], autophagy [15–17], angiogenesis [18–20], immunomodulation [21], invasion [14, 22, 23], and metastasis [24–26]. These pathways have been reviewed comprehensively elsewhere [27–31]. In addition to its effectiveness as an antineoplastic, curcumin has been found to be protective against reactive oxygen species (ROS). A study by Rathore et al. [32] examined the effects of curcuma oil on a transient ischemia model in Sprague-Dawley rats. The middle cerebral artery was occluded using a nylon filament for two hours, followed by a 24-hour reflow period. Treatment with curcuma oil reduced the size of infarcted cerebral tissue, improved neurologic function, and reduced oxidative stress caused during the reperfusion period. The authors reported that these protective effects were obtained by inhibition of ROS, reducing peroxynitrite levels and caspase-3 activity and preventing destabilization of the mitochondrial membrane potential [32]. In an* in vitro* model of ischemia-reperfusion using rat cortical neurons, curcumin was found to protect neurons from death caused by oxygen and glucose deprivation. The authors of this study suggested that curcumin activates an antioxidant protein, thioredoxin, in the Nrf2 pathway [33]. In addition, curcumin may inhibit cellular glyoxalases leading to decreased ATP and glutathione, which can impact cellular metabolism and may account for some of its anti-inflammatory and antitumor effects [34].

### 2.1. Curcumin Induces G2/M Cell Cycle Arrest

There is a bevy of data to suggest that curcumin is able to induce G2/M cell cycle arrest and apoptosis. More recent work has tried to explain how this cell cycle arrest can occur. Wu et al. published a study in 2013 [35] that suggests that this might be due to increased DAPK1 expression. The authors treated the U251 GBM cell line with curcumin and found a dose-dependent increase in DAPK1 mRNA by real-time RT-PCR and verified a corresponding increase in protein expression by western blot analysis. Further, they used siRNA (si-DAPK1-1 and si-DAPK1-2) transfection to suppress DAPK1 and found that curcumin had an attenuated ability to suppress STAT3 and NF-*κ*B phosphorylation. They also showed that their knockdown of DAPK1 inhibited curcumin-mediated caspase-3 activation and led to decreased apoptosis (33.0% apoptotic cells versus 58.3% in their control) [35]. These findings suggest that DAPK1 plays an important role in curcumin-mediated cell death. Other studies in U251 GBM cells show that p53 expression is upregulated by curcumin treatment, as were CDKi p21^Waf1/Cip1^ (p21) and ING4—a tumor suppressor gene that has been found to be suppressed in gliomas [36, 37]. Curcumin owes part of its antiproliferative effects to suppression of cyclin D1 and to induction of p21. In a study that treated U87 GBM cells with curcumin, transcription factor Egr-1 was found to activate transcription of p21 independent of p53 activation. Egr-1 expression was reportedly induced by curcumin via ERK and JNK [13]. An* in vitro* study using U87 human glioma cell line showed that 5–10 *μ*M curcumin could inhibit proliferation and that treated cells were arrested in G2/M stage by increased expression of DUSP-2 and inhibition of ERK and JNK phosphorylation. Interestingly, trapping cells in the G2/M phase appears to enhance their sensitivity to radiation [38, 39]. Curcumin's radiosensitizing effects have also been examined in neuroblastoma (SK-N-MC) cells. Pretreating cells with 100 nM curcumin suppressed radiation induced NF-*κ*B, enhanced radiation induced caspase activation, and inhibited antiapoptotic molecules [40].

### 2.2. Curcumin Activates Apoptotic Pathways

An* in vitro* study using human GBM (A172, KNS60, U251MG(KO)) and medulloblastoma (ONS76) cell lines showed that while curcumin was able to inhibit growth of these cell lines, only KNS60 and ONS76 were arrested at G2/M. These findings suggest that the mechanism for growth inhibition is not exclusively due to cell cycle arrest. They also found a significant increase in caspase 3/7 activity, curcumin induced DNA damage, and apoptosis triggered by overexpression of BAX and downregulating Bcl2 and survivin, as well as inhibition of telomerase and downregulation of hTERT [41]. An* in vitro* study using 8401 GBM cell line found that curcumin decreased cell proliferation, reduced mitochondrial membrane potential, induced DNA fragmentation, induced apoptosis via a caspase-dependent pathway (caspase-3, caspase-8, and caspase-9), and inhibited NF-*κ*B transcription factor activity [42]. In U87MG GBM cell line, curcumin can induce apoptosis by suppressing antiapoptotic signals, by promoting activation of caspase-8, and through an increased BAX/Bcl-2 ratio [43]. Solubilized curcumin (667 *μ*M in PBS, 3% DMSO) can increase caspase 3/7 activity in both mouse (B16F10 melanoma, GL261 glioma, and N18 neuroblastoma) and human (HOG oligodendroglioma and A549 lung cancer) cell lines and it decreased tumor cell viability as measured by MTT assay. Further, LDH release in these experiments was not increased, suggesting apoptotic cell death [19]. The authors of this study also report that cyclin D1, NF-*κ*B, AKT, ERK, and Bcl-xL were suppressed when B16F10 cells were treated with curcumin [19]. In an* in vivo* arm of this experiment, the authors injected 10,000 B16F10 mouse melanoma cells in the brain of C57BL/6 mice. They then received either daily tail vein injections of 200 *μ*L of their solubilized curcumin (estimated final plasma concentration of 35 *μ*M in PBS, 0.15% DMSO) or a control injection (PBS, 0.15% DMSO) for 18 days. Additionally, this experiment was repeated with intracerebral delivery of the curcumin solution via a stainless steel cannula. While all of the control animals developed intracranial tumors, only one of the 5 treated mice developed detectable tumor [19].

### 2.3. Curcumin Induces Autophagy

Autophagy is a cellular process for disassembling unnecessary or dysfunctional cellular components and is essential for maintaining energy during states of nutritional stress and during programmed cell death. It also appears to have an important role in regulation of glioma-initiating cells, which are poorly differentiated and share features of neural stem cells. An* in vitro* study published in 2012 showed that curcumin can induce differentiation and halt growth of glioma-initiating cells (SU-2 and SU-3) from surgically resected human GBM by activating autophagy. In the same study, glioma-initiating cells were implanted intracranially in athymic nude mice and randomized to a treatment group consisting of intraperitoneal (IP) injection of curcumin (300 mg/kg) every 3 days or control group (*n* = 14 each). Those mice who received treatment had increased survival (study halted after 120 days) versus nontreated animals [16]. In a separate study, curcumin activated the ERK1/2 pathway and inhibited the AKT/mTOR/p70S6K pathway, resulting in autophagy both* in vitro* and* in vivo* using a subcutaneous xenograft model in nude mice with U87 cells. The authors report that curcumin induced G2/M cell cycle arrest in addition to autophagy in U87 and U373 GBM cells [17].

### 2.4. Curcumin Disrupts Molecular Signaling

The NF-*κ*B pathway is upregulated in GBM, and inhibitors of NF-*κ*B exhibit relatively low toxicity to normal tissues. A preclinical study of NF-*κ*B inhibitors found that NF-*κ*B activity correlated with percentage cell viability in C6 and U138 GBM lines [45]. The authors report that inhibition of NF-*κ*B leads to mitochondrial dysfunction with arrest in the G2/M phase of the cell cycle. Interestingly, NF-*κ*B is found to be overstimulated in cisplatin-resistant C6 cells and NF-*κ*B inhibitors were able to overcome cisplatin resistance [45]. Similarly, progranulin has been found to be overexpressed in many GBM cell lines (U87, GBM8904, and S1R1) and tumor samples [46]. Progranulin overexpression contributes to tumorigenesis and treatment resistance by upregulating DNA repair and stemness genes using the transcription factor AP-1. Curcumin is an AP-1 inhibitor, and it was found to downregulate progranulin promoter activity and expression [46]. Other studies have shown that curcumin can reduce GBM cell survival through inhibition of AP-1 and NF-*κ*B by preventing constitutive activation of JNK and AKT [47]. An* in vitro* study examined the role of glucose-6-phosphate translocase (G6PT) in U87 GBM cell signal transduction. In this experiment, suppression of gene expression was accomplished using siRNAs and led to apoptosis and necrosis. Curcumin (35 *μ*M) also inhibited G6PT gene expression >90%. However, the authors also report transfection with an expression vector for G6PT rescued cells from curcumin-mediated cell death. This suggests that G6PT may be a novel chemotherapeutic target and that G6PT overexpression may lead to treatment resistance [48]. In a separate set of experiments, murine glioma cell lines (Tu-2449, Tu-9648, and Tu-251) with constitutively expressed STAT3 treated with curcumin showed a dose-dependent decrease in the activity of phosphorylated JAK1 and JAK2 and led to downstream inactivation of STAT3. In an* in vivo* arm of this study, C6B3F1 mice were fed a diet rich in fat and cholesterol with or without fortification with curcumin for 7 days. Subsequently, they received intracranial implantation of either Tu-2449 or Tu-9648 glioma cells and maintained their diet. Fifteen percent of Tu-2449 implanted mice who received curcumin-enriched diets had tumor-free long-term survival versus 0% of the control dieted animals. Curcumin fed Tu-9648 implanted mice experienced a 38% increase in tumor-free long-term survival [49].

The sonic hedgehog (SHH) signaling pathways are important in the carcinogenesis of medulloblastoma. It is a major regulator of cell proliferation and death. Curcumin has been shown to downregulate SHH, leading to decreased downstream targets GLI1 and PTCH1 and cytotoxicity in cell lines MED-4, MED-5, and DAOY [50]. Targeting SHH and GLI1 with curcumin treatment has also been done in glioma cells (U87 and T98G). Similar to medulloblastoma, mRNA and protein levels of SHH and GLI1 were downregulated with curcumin treatment. IP injection of curcumin in U87-implanted nude mice also reduced tumor volume, prolonged survival, and decreased GLI1 expression [51]. In the medulloblastoma cell line DAOY, curcumin has been shown to inhibit Wnt/*β*-catenin signaling and suppress proliferation with IC_50_ of 35 *μ*M after 48 hours [52]. Mechanistically, curcumin may decrease histone deacetylase 4 expression leading to increased tubulin acetylation and mitotic catastrophe in addition to its effects on G2/M cell cycle arrest in DAOY medulloblastoma cells [53].

### 2.5. Curcumin Decreases Invasion and Metastasis

Abnormal expression of matrix metalloproteinases (MMPs), membrane-associated or secreted enzymes used to digest extracellular matrix proteins, is one method by which glioma cells are able to invade normal brain tissue. Data has shown that curcumin is able to suppress expression of MMP-1, MMP-3, MMP-9, and MMP-14 in GBM cell lines U87MG and U373MG via the common upstream AP-1 and MAP kinases [22, 23, 54]. Urokinase-type plasminogen activator (uPA) is a serine protease that starts a degradative cascade by converting extracellular plasminogen to plasmin and eventually degrades extracellular matrix collagen and activates other MMPs to aid in invasion [55]. Curcumin can prevent nuclear translocation of RelA/NF-*κ*B, which prevents upregulation of uPA [55]. Curcumin has also been found to dramatically reduce MMP-9 in murine glioma cell lines Tu-2449, Tu-9648, and Tu-251 [49]. An important contributor to tumors propensity to invade and metastasize is angiogenesis. Cell lines derived from surgically resected pituitary adenomas treated with curcumin have decreased HIF1A and VEGFA, both of which are involved in tumoral angiogenesis [56].

### 2.6. Curcumin Increases the Efficacy of Existing Chemotherapeutics

Data also exists to suggest that curcumin acts synergistically with chemotherapeutics already approved for the treatment of brain tumors. A preclinical study examined the efficacy of curcumin for the treatment of GBM* in vitro* and* in vivo* [57]. Cell lines U138MG, U87, U373, and C6 were treated with curcumin and found to have IC_50_ values of 29, 19, 21, and 25 *μ*M, respectively, whereas IC_50_ for astrocytes was 135 *μ*M. Chemotherapy synergism was subsequently tested using U138 and C6 cell lines. Curcumin alone resulted in 55% and 75% viability, respectively. When cisplatin was added in combination with 25 *μ*M curcumin, the viability dropped to 30% and 10% for U138 and C6 cell lines, respectively. When doxorubicin was added in combination with 25 *μ*M curcumin, viability was reported to be 36% and 46%. Mechanistically, the authors reported that curcumin decreased activation of PI3K/AKT and NF-*κ*B pathways, decreased expression of Bcl-xL, and caused mitochondrial dysfunction and apoptosis. The authors also implanted Wistar rats with C6 GBM cells intracranially and treated them with 50 mg/kg/day curcumin via IP injection within days 10–20 after implantation. Sixty-four percent of curcumin treated animals developed tumors as compared to 100% of the DMSO treated controls. Of the treated animals who developed tumors, average tumor volume was 73% less than that of the controls [57]. Curcumin in combination with temozolomide (TMZ) appears to have additive cytotoxic benefit in GBM cells [15]. Further, both drugs appear to cause G2/M arrest by activating proteins such as Wee, Cdc2, CHK1, and Cdc25c. Decreased phosphorylation of cyclin B1 and cyclin D1 was also observed [15]. The authors found that treatment with either TMZ or curcumin appears to induce autophagy that was dependent on ERK1/2 prior to apoptosis. Curcumin was also found to inhibit STAT3, NF-*κ*B, and PI3K/AKT [15]. A study by Ramachandran et al. [58] investigated potentiation of etoposide (ETP) and TMZ by curcumin and a trade preparation of turmeric called Tumeric Force™ (TF) in human U87 GBM and D283 medulloblastoma cell lines. U87 IC_50_ values for ETP, TMZ, curcumin, and TF were 6.5, >2000, 37.3, and 30.8 *μ*g/mL, respectively. For D283 cells, those IC_50_ values were 0.19, 147, 28.7, and 1.6 *μ*g/mL. Combination index values in U87 glioma cells for ETP + curcumin, TMZ + curcumin, and ETP + TMZ + curcumin were 0.55, 2.07, and 0.39 *μ*g/mL, respectively. The combination index values with TF instead of curcumin were 0.38, 0.57, and 0.05 *μ*g/mL [58]. Curcumin has also been studied for efficacy in combination with paclitaxel (PTX) for the treatment of GBM. LN18 and U138MG cell lines treated with 20 *μ*M curcumin and 10 nM PTX were found to have a combination index of 0.1 and 0.09, respectively, indicating a synergistic effect. This combination activated caspase-3, caspase-8, and calpain, increased BAX, and reduced Bcl-2 initiating apoptosis. Combination therapy also decreased the ability of LN18 and U138MG cells to invade matrigel [59]. In addition to acting synergistically, curcumin may prevent chemotherapy resistance. A study reported in 2008 on Sprague-Dawley rats showed curcumin can inhibit a protein linked with multidrug resistance in the luminal membrane of capillaries at the blood-brain barrier- (BBB-) ATP-binding cassette transporter ABCG2 [60]. A separate, intriguing method by which GBM may resist hydrophobic therapeutic agents is by accumulating lipid droplets that sequester these drugs. Curcumin is hydrophobic and has been shown to concentrate near the cell membrane and in cytoplasmic droplets of U251N human GBM cells [61]. Zhang et al. sought to overcome this mechanism of resistance* in vitro* using pyrrolidine-2, a cytoplasmic phospholipase A2*α* inhibitor, in combination with curcumin. When the cells were pretreated with pyrrolidine-2 24 hours before curcumin administration, cell viability was found to approach 0% [61].

### 2.7. Curcuminoids and Curcumin Derivatives

Demethoxycurcumin (C2) and bisdemethoxycurcumin (C3) (Figure 1) are two curcuminoids often found in small percentages of curcumin extractions. An* in silico* study published in 2009 [62] examined the docking of curcuminoids with the Bcl-2 apoptotic proteins (Protein Data Bank accession numbers 1G5M and 1GJH). The authors calculated free energies and inhibition constants for these molecules and found that C2 binds more favorably than curcumin (Δ*G*  −6.97 versus −4.53 kcal/mol; *K*_*i*_ 0.56 versus 2.21 nm).* In vitro* treatment of U87 GBM cells showed marked decrease in Bcl-2 expression with all three compounds after 48 hours and that C2 showed significant progression of percent apoptosis compared to curcumin or C3. An* in vitro* binding assay utilizing circular dichroism spectroscopy showed that C2, like the natural Bcl-2 inhibitor Bak, caused a conformational change different from curcumin or C3 [62]. These findings suggest that C2 induces Bcl-2 mediated apoptosis more effectively than curcumin or C3. A study published by Huang et al. [63] also examined the effects of C2. They showed that C2 had IC_50_ of 22.7 *μ*M in GBM 8401 cells and that apoptosis was induced by decreasing the mitochondrial membrane potential and caspase-dependent pathways similar to other studies of curcumin [63]. Other curcumin analogs have been developed in an attempt to improve upon the compound's therapeutic promise. For example, lead compounds identified by Campos et al. show comparable IC_50_ values against GBM (U87MG) and neuroblastoma (SK-N-SH and SK-N-FI) cell lines [64].

## 3. Delivery Mechanisms

The therapeutic benefits of curcumin are limited by its poor absorption, rapid metabolism, and poor water solubility [65]. Curcumin is broken down by multiple enzymatic pathways, including glucuronidation, sulfation, alcohol dehydrogenase, and p450 system leading to rapid metabolism and excretion [66]. However, its lack of toxicity allows for administration of large doses. Twenty-four subjects with mean age of 34 were provided capsules with 0.5–12 g of curcumin and serum analysis was conducted prior to testing at 1, 2, and 4 hours after dosing. Peak serum concentration was <2 *μ*M even at 10- and 12-gram doses [67, 68]. These clinical data also show that doses up to 12 grams per day do not provoke serious side effects, though trouble with tablet bulk, diarrhea, and yellow stool were noted [67, 68]. Piperine, an inhibitor of hepatic and intestinal glucuronidation, has long been suggested to be administered as an adjuvant to prevent the degradation of medications including curcumin [69–71]. Ten subjects with mean age of 60 years participated in a randomized crossover trial to compare the effects of oral curcumin (2 g) alone or with the addition of piperine (20 mg). A two-week washout period was used before the crossover. They found that, when administered with piperine, peak serum concentrations of curcumin increased 30-fold and the relative bioavailability was 2000%. The *V*_*D*_ for curcumin administered with piperine was 203 L/kg. No adverse events were reported in this study [69].

Though current literature contains mixed data, curcumin distribution to the brain might be hindered by limited BBB permeability [65, 72]. Certain nanoparticle formulations, such as poly(lactic-co-glycolic acid), have been shown to increase distribution to brain tissue [65]. The half-life of curcumin-loaded poly(lactic-co-glycolic acid) in brain tissues from Sprague-Dawley rats for this study was reported to increase from 9 minutes to 15 minutes. When brain regions were examined separately, the compound's half-life was found to be significantly extended in the cerebral cortex (20 versus 2 minutes) and hippocampus (17 versus 8 minutes) but not in the cerebellum, brainstem, striatum, or other brain regions [65]. Curcumin has also been loaded into poly(butyl)cyanoacrylate (PBCA) nanoparticles using apolipoprotein E3 to increase transport across the BBB [73]. This delivery system was found to induce apoptosis in SH-SY5Y neuroblastoma cells more efficaciously than curcumin solution or curcumin loaded in the PBCA carrier without ApoE3 [73]. Nanostructured lipid carriers (tripalmitin-oleic acid) loaded with curcumin had IC_50_ of approximately 20 *μ*g/mL against A172 GBM cells compared to IC_50_ of 80 *μ*g/mL with curcumin alone. Further, the authors showed that curcumin delivered with the lipid carriers to subcutaneous flank tumor bearing nude mice decreased tumor volume by 82% [74]. Polymeric micelles have also been studied for curcumin delivery. Amphiphilic block copolymers form micelles when placed in aqueous solutions. These vesicles, or polymersomes, can be made of one or more different high-molecular weight amphiphilic block copolymers such as oleic acid and PEG 400 as a diblock nanostructure. This combination has been shown effective in delivering curcumin and reducing tumor burden in mouse models [75, 76]. Monomethoxy PEG has a higher molecular weight of 2 kDa versus 400 Da and has also been tested in attempt to obtain a better thermodynamic profile [77]. When tested* in vitro* with the U87 human glioblastoma cell line, monomethoxy PEG-oleate conjugation product showed IC_50_ values of 24 *μ*M versus IC_50_ of 48 *μ*M for free curcumin [77]. Intranasal delivery of therapeutics to the brain represents a noninvasive way to bypass the BBB. Exosome encapsulated curcumin has been administered intranasally to C57BL/6j mice in an attempt to treat inflammation induced by administration of lipopolysaccharide (LPS) [78]. It was found that these exosomes were taken up by brain microglia within 15 minutes and inhibited LPS-induced inflammation [78]. Other lipid carriers have also been designed for intranasal curcumin delivery to the central nervous system. Testing in Wistar rats showed that maximum concentration was reached after approximately 3 hours, and delivery to brain tissues was greater with the lipid carrier than the plain drug suspension (86 and 54 ng/g, resp.) [79].

Dendrosome carriers, comprised of esterified oleoyl chloride and polyethylene glycol 400, have recently been published as effective means of delivery curcumin [75, 76].* In vitro* data treating U87MG GBM cells with curcumin, dendrosomal curcumin, and empty dendrosome found that dendrosomal curcumin had superior efficacy at 24, 48, and 72 hours. As with many other studies, the authors found increased caspase activity when these cells were treated with curcumin. Interestingly, they also examined pluripotency transcription factors (OCT4A, OCT4BI, SOX-2, and Nanog) and found that treating U87 cells with dendrosomal curcumin led to significant decreases in these factors. During cellular differentiation, miR-145 expression suppresses these pluripotent genes. The authors reported that dendrosomal curcumin was able to exert its effects by inciting an approximately 35-fold elevation of miR-145 [75]. Treatment of A431 (epidermoid carcinoma) and WEHI-164 (mouse fibrosarcoma) cell lines with dendrosomal curcumin resulted in IC_50_ values of 14.3 and 7.5 *μ*M at 48 hours, respectively, compared to 37 *μ*M for curcumin alone against WEKI-164 cells [76]. WEHI-164 cells were injected subcutaneously in the flank in an* in vivo* experiment using BALB/c mice, and the results showed that treatment with dendrosomal curcumin led to significant reductions in tumor burden compared to curcumin treated animals or control [76]. Dendrosomal curcumin has also shown efficacy in the treatment of metastatic breast cancer cells (4T1)* in vitro* and* in vivo* with 11% of animals having metastases at necropsy compared to 89% of control. The authors also reported that treatment with dendrosomal curcumin led to downregulation of VEGF, COX-2, and MMP expression [80].

Curcumin has also been conjugated as a poly(glycerol-sebacate-curcumin) polymer similar to carmustine polymers used for local treatment with gliomas [81].* In vitro* studies show IC_50_ values in response to this polymer of 23.2 *μ*g/mL with U87 cells and 20.2 *μ*g/mL with T98 cells [81]. Polycaprolactone implants have been studied as another delivery method for test agents with poor bioavailability. An* in vitro* assay with 9 L rat glioma cells reported that curcumin-loaded poly(ɛ-caprolactone)-poly(ethylene glycol)-poly(ɛ-caprolactone) nanofibers caused dose-dependent growth inhibition [82]. Gupta et al. reported a preclinical study [83] in August-Copenhagen Irish rats who received four polycaprolactone implants that were loaded with 40 mg of curcumin. Analysis of liver, brain, and plasma by high-performance liquid chromatography (HPLC) with fluorescence spectroscopy showed an increase in curcumin levels versus animals receiving sham implant and a diet supplemented with 1000 ppm curcumin [83]. They also demonstrated that these implants can reduce benzo[a]pyrene induced lung DNA adducts by 62% in Sprague-Dawley rats [83].

Curcumin microparticles in a poly(D,L-lactide-co-glycolide) polymer has been formulated in an attempt to improve delivery of the drug and sustain its release. The investigators reported that a single subcutaneous injection was able to sustain release in the blood and body tissues of BALB/c mice for 4 weeks. Levels of curcumin in the lungs and brain show 10–30-fold greater distribution than the blood. Further, animals implanted with MDA-MB-231 human breast adenocarcinoma cells were effectively treated with these microparticles (49% decreased tumor volume versus empty microparticle), whereas repeated systemic injections of curcumin alone were no different than the vehicle treatment [84]. Curcumin nanoparticles (NanoCurc™) [21, 85] have also been used as a method to overcome the delivery and bioavailability barriers of curcumin. These particles, unlike free curcumin, are able to disperse in an aqueous environment [21]. Studies using xenograft models of human pancreatic cancer in athymic mice show that NanoCurc™ can lead to approximately 50% reduction in tumor volume. This inhibition was enhanced with the addition of gemcitabine. The authors reported that these effects were observed through decreased NF-*κ*B activation, MMP-9, and cyclin D1 [85]. These nanoparticles have also been shown to suppress* in vitro* growth by MTS cell proliferation assay of embryonal tumor lines (DAOY and D283Med), U87 GBM cells, and glioblastoma neurosphere lines (JHH-GBM14 and HSR-GBM1) in a dose-dependent manner [86]. The authors suggested that, in this experiment, the effects could be due to a combination of G2/M arrest and apoptotic induction [86].

Additional benefits of using delivery systems include the ability to deliver multiple compounds simultaneously. For example, Dilnawaz and Sahoo used magnetic nanoparticles (MNP) to deliver both curcumin and TMZ to monolayer and spheroid T-98G GBM cultures. In monolayer cultures, IC_50_ values (*μ*g/mL) for TMZ, curcumin, TMZ and curcumin were 1.1, 6.6, and 0.8 without MNP delivery and 0.2, 0.6, and 0.1 with MNP delivery. These same values in spheroid cultures were 23, 28, and 10 without MNP and 10, 11, and 5.2 with MNP delivery. Combination index analysis showed that the drug combination functioned synergistically in both cultures [87]. Another study using magnetic core nanocapsules reported the ability to deliver drugs with differing hydrophobicity [88]. They were able to accommodate doxorubicin (hydrophilic) and curcumin (hydrophobic) in the same particles for delivery to RG2 rat glioma cells [88].

Curcumin has also been coupled to an antibody as a delivery vehicle. Langone et al. [89] demonstrated that curcumin coupled to Muc18, a melanoma specific antibody, increased its efficacy against B16F10 melanoma. The* in vitro* IC_50_ value was reduced from 22 *μ*M to 0.09 *μ*M in cells treated with the curcumin-antibody adduct versus curcumin alone. In addition, C57BL/6 mice implanted with 1,000 B16F10 cells in their right forebrain showed a 10-fold decrease in tumor burden when treated with and curcumin-antibody adduct [89]. The authors reported that this effect was achieved by suppressing NF-*κ*B [89]. In 2014, Langone et al. published another study that used an antibody delivery system for curcumin. In this experiment, a GBM-specific CD68 antibody was used to target GL261 (mouse), T98G (human), and U87MG (human) GBM cells [18]. GL261 cells were treated* in vitro* with 50 *μ*M of curcumin for 24 hours, and western blot analysis showed suppression of NF-*κ*B. In addition, curcumin was found to inhibit AKT-1 (neural survival and antiapoptotic), Bcl-xL (antiapoptotic), cyclin D1 (cell cycle promoter), and VEGF (promoting angiogenesis) [18]. Curcumin alone yielded IC_50_ 15 *μ*M, whereas treatment with the curcumin-antibody adduct had IC_50_ of 0.125 *μ*M. IC_50_ values for U87MG and T98G cells were 25 and 8 *μ*M for curcumin alone and 0.400 and 0.225 *μ*M, respectively, for treatment with the CD68 antibody-linked curcumin. GL261 cells were also implanted in the right forebrain of C57BL6 mice. These mice were treated with intracranial infusions of 16 pmol CD68 antibody-linked curcumin followed by tail vein infusion of curcumin and resulted in a reduction of intracranial tumor burden [18].

## 4. Toxicity/Safety

Natural products are of interest as anticancer agents because they have been associated with low toxicity profiles. This allows them to be safely used at high doses or added to existing regimens. Indeed, curcumin has been administered to human subjects at large doses without major side effects [67–69]. However, some data exists that serves to caution against its use. Despite being an antioxidant, curcumin may lead to a temporary increase in ROS and a decrease in cell viability by depletion of glutathione [90]. Curcumin might cause DNA base damage and fragmentation through cytochrome p450 enzyme generation of a curcumin radical and lead to apoptotic cell death in healthy as well as tumoral tissues [91]. Interestingly, this damage was attenuated when curcumin was present at larger concentrations [92]. Similar DNA damage findings have been noted during* in vivo* studies [93, 94]. Curcumin may inhibit the tumor suppressor p53 function in colon cancer cells and contribute to tumorigenesis [95]. Administration of curcumin with camptothecin or cyclophosphamide may inhibit their effectiveness; thus careful study of curcumin is warranted prior to its addition to an existing chemotherapy [96].

## 5. Clinical Data

There are over one hundred clinical trials examining the potential therapeutic effects of curcumin (Table 1, https://clinicaltrials.gov/). Many of these trials examine the efficacy of curcumin for the treatment of gastrointestinal diseases (e.g., Cohn's disease, ulcerative colitis, and inflammatory bowel disease), endocrine disorders (e.g., diabetes mellitus), neoplasms (breast, gastrointestinal, cervical, lymphoma, and pancreatic), immune system diseases (e.g., atopy, multiple sclerosis, and rheumatoid arthritis), and psychiatric disorders (e.g., Alzheimer's disease, schizophrenia, cognitive impairment, and depression). To date, no clinical data exist on treatment of brain tumors with curcumin despite the vast amount of promising preclinical data. A study completed in May 2013 (NCT01712542) examined the bioavailability of curcumin in patients with GBM, but results from this study have not yet been made available. Other human bioavailability data are discussed in “Delivery Mechanisms.”

## 6. Conclusion

The use of curcumin for the treatment of CNS tumors needs to be investigated. Preclinical data support its use* in vitro* and* in vivo*. In addition, there have been several limited studies demonstrating the safety of administration of oral curcumin in humans. Initial efficacy studies might focus on patients that have progressed on the current standard of care. As discussed previously, curcumin may also enhance the cytotoxic capabilities of other chemotherapeutics. Data also exists to suggest that curcumin might affect tumoral stem cell-like populations [16, 46, 97]. These stem cell-like features are found in nestin and CD133 positive cells are associated with higher grade and poor prognosis [46, 98, 99]. Adding curcumin to other chemotherapeutic regimens may aid in slowing or halting growth of these progenitor cells and thus their tumors. Since the administration of curcumin appears relatively safe, it could be added to current standard of care regimens in early or advanced disease. The greatest barrier to the use of curcumin as a therapeutic is its poor distribution to affected tissues. The use of delivery vehicles or curcumin derivatives may increase the efficacy of curcumin further by improving distribution, slowing degradation, and improving target specific affinity. Since individuals afflicted with brain tumors have such poor prognoses, there is a dire need to identify new therapeutic agents. As in the case with GBM, it is unfortunate that large clinical trials have been undertaken without identifying improvements to the standard of care that was formulated over 10 years ago [100–104]. Clinical trials should be undertaken to corroborate the benefits of curcumin seen in preclinical studies and improve the prognoses of individuals with brain tumors.

## Figures and Tables

**Table 1:** Active clinical trials assessing therapeutic benefit of curcumin.

Trial	Status	Site	Disease	Measure	Trial ID^*∗*^
*Vascular*					
Curcumin or placebo	Ongoing	Lawson Health Research Institute, CA	AAA	Serum creatinine	NCT01225094
Curcumin or placebo	Ongoing	University of Colorado Boulder, USA	Vascular aging	Arterial stiffness, arterial pulse-wave velocity, NO dilation	NCT01968564
Curcumin or placebo	Ongoing	UPEI, CA	Vascular stiffness	Arterial stiffness by tonometry, IL-6, CRP, CK	NCT02281981

*Neurologic/psychiatric*					
Curcumin with or without yoga	Recruiting	VA Los Angeles, USA	AD	Biomarkers for MCI	NCT01811381
Curcumin	Recruiting	SHSC, CA	Adolescent bipolar disorder	Mood by CDRS-R, biomarkers	NCT01928043
Curcumin with other supplements or placebo	Ongoing	HCL, France	Fibromyalgia	QOL by GIQLI	NCT01469936
Curcumin or placebo	Ongoing	UCLA, USA	MCI, normal aging	Cognitive changes by neuropsych assessment	NCT01383161
Curcumin or placebo with omega-3 fatty acid	Invitation only	TUMS, Iran	Migraine	HA, endothelial factors, inflammation	NCT02532023
Curcumin or placebo with IFN-*β*1A	Ongoing	Merck Serono, Italy	Multiple sclerosis	Proportion of subjects with active T2 lesions	NCT01514370
Curcumin or placebo	Recruiting	Beersheva Mental Health Center, Israel	Schizophrenia	Psychotic symptoms (PANSS)	NCT02298985
Curcumin	Recruiting	VA Los Angeles, USA	Schizophrenia	MCCB	NCT02104752
Curcumin or placebo	Not yet recruiting	Yale, USA	Schizophrenia, schizoaffective	MCCB	NCT02476708

*Gastrointestinal*					
Curcumin or placebo with thiopurines	Recruiting	CHU, France	Crohn's disease	Rutgeerts endoscopic score	NCT02255370
Curcumin with triple therapy or triple therapy alone	Not yet recruiting	Rabin Medical Center, Israel	Helicobacter pylori infection	Eradication by urea breath test	NCT02018328
Curcumin with selenium and green tea or placebo	Recruiting	Meir Medical Center, Israel	Irritable bowel syndrome	QOL by questionnaires	NCT01167673
Curcumin or placebo	Not yet recruiting	NCI, USA	MAG and/or GIM	Changes in IL-1*β*, safety, tolerability, histologic grade	NCT02782949
Curcumin or placebo	Not yet recruiting	Schneider Children's Medical Center, Israel	Pediatric ulcerative colitis	Disease activity by PUCAI	NCT02277223
Curcumin or placebo with 5-ASA	Recruiting	Asian Institute of Gastroenterology, India	Ulcerative colitis	Time to clinical and endoscopic remission	NCT02683733
Curcumin or placebo with 5-ASA	Recruiting	Asian Institute of Gastroenterology, India	Ulcerative colitis in remission	Percentage of patients in remission	NCT02683759

*Oncologic*					
Curcumin or placebo	Recruiting	Emory University, USA	Breast cancer	NF-*κ*B by ELISA	NCT01740323
Curcumin at two different doses	Recruiting	OSU, USA	Breast cancer, obesity	Adherence, tolerability, safety	NCT01975363
Curcumin or HPR Plus or placebo	Recruiting	University of Rochester, USA	Noninflammatory breast cancer	Mean radiation dermatitis severity	NCT02556632
Curcumin with standard treatment	Recruiting	UZ Leuven, Belgium	Endometrial carcinoma	Peripheral blood inflammatory markers	NCT02017353
Curcumin	Recruiting	Baylor Research Institute, USA	Squamous CIN3	Safety, feasibility, overall and pathologic response	NCT02554344
Curcumin and piperine	Recruiting	Mayo, USA	Cancer with inflamed ureteral stent	AE, max tolerable dose, optimal dose	NCT02598726
Curcumin with cholecalciferol	Recruiting	Case CCC, USA	CLL or SLL, stages 0-II	Overall response rate by NCI-WG (CLL) or Cheson (SLL)	NCT02100423
Curcumin	Recruiting	University Hospital Salzburg, Austria	Locally advanced or metastatic cancer	Safety, max tolerable dose, tumor response	NCT02138955
Curcumin or placebo	Recruiting	UPR, Puerto Rico	FAP	Tolerability and efficacy by polyp number and size	NCT00927485
Curcumin or placebo	Ongoing	NCI, USA	FAP	Laboratory biomarker analysis	NCT00641147
Curcumin with anthocyanin extract	Recruiting	The Hospital Galliera, Italy	Colorectal adenoma	*β*-Catenin, NFk*β*, Ki-67, p53 by IHC	NCT01948661
Curcumin with 5-FU	Recruiting	Baylor Research Institute, USA	Metastatic colon cancer resistant to 5-FU	Safety, toxicity, response, biomarkers	NCT02724202
Curcumin with FOLFOX or FOLFOX alone	Ongoing	University of Leicester, UK	Metastatic colorectal cancer	Tolerable long-term dose, safety	NCT01490996
Curcumin with Avastin/FOLFIRI	Not yet recruiting	Gachon University Gil Medical Center, Korea	Metastatic colorectal cancer	PFS	NCT02439385
Curcumin with irinotecan	Recruiting	UNC Lineberger, USA	Metastatic colorectal cancer	Max tolerated dose, pharmacokinetics	NCT01859858
Curcumin or placebo with capecitabine and radiation	Ongoing	MD Anderson, USA	Rectal cancer	Pathologic complete response rate	NCT00745134
Curcumin or placebo with docetaxel	Recruiting	Centre Jean Perrin, France	Metastatic prostate cancer	Time to progression, PSA response	NCT02095717
Curcumin or placebo	Recruiting	UT Southwestern, USA	Prostate cancer	PSA, recurrence free survival	NCT02064673
Curcumin or placebo with RT	Recruiting	SBUMS, Iran	Prostate cancer	Proctitis and cystitis by CTCAE, PSA	NCT02724618
Curcumin with gemcitabine, metformin, and paclitaxel	Recruiting	City of Hope Medical Center, USA	Metastatic pancreatic cancer	Feasibility, compliance, toxicity, survival	NCT02336087
Curcumin with EGFR-TKI	Recruiting	Lady Davis Institute, CA	Nonresectable mutant EGFR NSCLC	Feasibility, adherence, AE, QOL (FACT-L), CRP	NCT02321293

*Other*					
Curcumin or placebo	Recruiting	University of Colorado Denver, USA	ADPKD	Changes in FMD-BA and aortic pulse-wave velocity	NCT02494141
Curcumin mouthwash	Recruiting	Aurora BayCare Medical Center, USA	Chemotherapy induced mucositis	AE, toxicity, pain, healing time	NCT02300727
Curcumin or placebo	Recruiting	Lawson Health Research Institute, CA	CKD	Albuminuria, eGFR, IL-18	NCT02369549
Curcumin or placebo	Recruiting	NNFTI, Iran	DMII	Triglyceride and CRP levels	NCT02529969
Curcumin or placebo	Recruiting	NNFTI, Iran	DMII	Fasting blood sugar, antioxidant capacity	NCT02529982
Curcumin with other nutraceuticals or placebo	Not yet recruiting	IRCCS Neuromed, Italy	NAFLD	ALT, AST, GGT	NCT02369536
Curcumin in Orabase	Ongoing	SVSIDS, India	Oral submucous fibrosis	Reduction of lesion, number of bands	NCT02645656
Curcumin	Recruiting	University of Arizona, USA	Rheumatoid arthritis	AE, pharmacokinetics, ESR, CRP	NCT02543931

^*∗*^ *Source*: https://www.clinicaltrials.gov/. A search performed using the keyword “curcumin” revealed 129 studies. Only active studies (48) were included in the table. Clinical trials that were complete (58), were withdrawn (8), were terminated (3), or have unknown status (12) were excluded.

CA: Canada; CCC: Comprehensive Cancer Center; CHU: Clermont-Ferrand University Hospital; HCL: Hospices Civiles de Lyon; NCI: National Cancer Institute; NNFTI: National Nutrition and Food Technology Institute; OSU: Ohio State University; SBUMS: Shahid Beheshti University of Medical Sciences; SHSC: Sunnybrook Health Sciences Centre; SVSIDS: Sri Venkata Sai Institute of Dental Sciences; TUMS: Tehran University of Medical Sciences; UCLA: University of California, Los Angeles; UK: United Kingdom; UNC: University of North Carolina; UPEI: University of Prince Edward Island; UPR: University of Puerto Rico; UT: University of Texas; USA: United States of America.5-ASA: 5-Aminosalicylic Acid; 5-FU: 5-Fluorouracil; AAA: Abdominal Aortic Aneurysm; AD: Alzheimer's disease; ADPKD: Autosomal Dominant Polycystic Kidney Disease; AE: adverse events; ALT: Alanine Aminotransferase; AST: Aspartate Aminotransferase; CDRS-R: Children's Depression Rating Scale-Revise; CIN: Cervical Intraepithelial Neoplasia; CK: Creatine Kinase; CKD: Chronic Kidney Disease; CLL: Chronic Lymphocytic Leukemia; CRP: C-Reactive Protein; DMII: Diabetes Mellitus Type 2; EGFR: epidermal growth factor receptor; eGFR: Estimated Glomerular Filtration Rate; ESR: Erythrocyte Sedimentation Rate; FAP: Familial Adenomatous Polyposis; FMD-BA: Brachial Artery Flow-Mediated Dilation; FOLFOX: Folinic Acid, 5-Fluorouracil, Oxaliplatin; GGT: Gamma-Glutamyl Transferase; GIM: Gastric Intestinal Metaplasia; GIQLI: Gastrointestinal Quality of Life Index; HA: headache; IHC: Immunohistochemistry; IL: Interleukin; MAG: Multifocal Atrophic Gastritis; MCCB: MATRICS Consensus Cognitive Battery; MCI: Mild Cognitive Impairment; NCI-WG: National Cancer Institute-Working Group; NAFLD: Nonalcoholic Fatty Liver Disease; NF-*κ*B: nuclear factor kappa-light-chain-enhancer of activated B cells; NO: Nitric Oxide; NSCLC: Nonsmall Cell Lung Cancer; PANSS: Positive and Negative Syndrome Scale; PFS: Progression Free Survival; PSA: prostate-specific antigen; PUCAI: Pediatric Ulcerative Colitis Activity Index; QOL: quality of life; RT: Radiotherapy; SLL: Small Lymphocytic Lymphoma; TKI: Tyrosine Kinase Inhibitor; VA: Veterans Affairs.
